# Non-Cytotoxic Quantum Dot–Chitosan Nanogel Biosensing Probe for Potential Cancer Targeting Agent

**DOI:** 10.3390/nano5042359

**Published:** 2015-12-18

**Authors:** Tyler Maxwell, Tahmina Banu, Edward Price, Jeremy Tharkur, Maria Gabriela Nogueira Campos, Andre Gesquiere, Swadeshmukul Santra

**Affiliations:** 1 Department of Chemistry, University of Central Florida, 4000 Central Florida Blvd., Orlando, FL 32816, USA; E-Mails: tyler.maxwell@knights.ucf.edu (T.M.); eprice2@knights.ucf.edu (E.P.); 2 NanoScience Technology Center, University of Central Florida, 12424 Research Parkway, Suite 400, Orlando, FL 32826, USA; E-Mails: tahminabanu@knights.ucf.edu (T.B.); jeremyt@knights.ucf.edu (J.T.); maria.nogueiracampos@ucf.edu (M.G.N.C.); 3 Department of Material Science and Engineering, University of Central Florida, 127600 Pegasus Drive, Engineering 1, Suite 207, Orlando, FL 32816, USA; 4 Burnett School of Biomedical Sciences, University of Central Florida College of Medicine, 6900 Lake Nona Boulevard, Orlando, FL 32827, USA; 5 Institute of Science and Technology, Federal University of Alfenas, Rodovia José Aurélio Vilela, 11999, Poços de Caldas, MG 37715-400, Brazil; 6 College of Optics and Photonics, University of Central Florida, P.O. Box 162700, Orlando, FL 32816, USA

**Keywords:** quantum dot, biosensor, chitosan, drug delivery, cancer, nanogel

## Abstract

Quantum dot (Qdot) biosensors have consistently provided valuable information to researchers about cellular activity due to their unique fluorescent properties. Many of the most popularly used Qdots contain cadmium, posing the risk of toxicity that could negate their attractive optical properties. The design of a non-cytotoxic probe usually involves multiple components and a complex synthesis process. In this paper, the design and synthesis of a non-cytotoxic Qdot-chitosan nanogel composite using straight-forward cyanogen bromide (CNBr) coupling is reported. The probe was characterized by spectroscopy (UV-Vis, fluorescence), microscopy (Fluorescence, Scanning Electron Microscopy (SEM), Transmission Electron Microscopy (TEM) and Dynamic Light Scattering. This activatable (“OFF”/“ON”) probe contains a core–shell Qdot (CdS:Mn/ZnS) capped with dopamine, which acts as a fluorescence quencher and a model drug. Dopamine capped “OFF” Qdots can undergo ligand exchange with intercellular glutathione, which turns the Qdots “ON” to restore fluorescence. These Qdots were then coated with chitosan (natural biocompatible polymer) functionalized with folic acid (targeting motif) and Fluorescein Isothiocyanate (FITC; fluorescent dye). To demonstrate cancer cell targetability, the interaction of the probe with cells that express different folate receptor levels was analyzed, and the cytotoxicity of the probe was evaluated on these cells and was shown to be nontoxic even at concentrations as high as 100 mg/L.

## 1. Introduction

Quantum dots (Qdots) are small crystals made of semiconductor material that possess unique size dependent optical properties due to confinement of their electronic states [1,2,3,4]. Luminescent Qdots are photo-stable compared to traditional organic dies, which has led to their study for *in vitro* and *in vivo* imaging [2,5,6]. A core–shell structure is commonly employed in Qdot synthesis to achieve high quantum yield, and to lower the toxic effects of cadmium [1,5,7,8]. Qdots have a broad excitation and tunable emission spectra, which allows for multiplexed imaging [9,10,11]. Targeted delivery of Qdots to cells for sensing and imaging applications has been refined in recent years by attaching antibodies, proteins, and other small molecules to the Qdot surface for highly sensitive whole body imaging and detection. These nanoparticles have also been employed for multimodal imaging by attachment of magnetic particles for MRI [8,12,13,14] and optical imaging [15] of tumor cells. Previous studies have shown that Qdots show promise as vehicles for the delivery of drugs as they can be conjugated with multiple functionalities for targeted delivery and imaging of drug release events [5,6,9].

Currently, there has been much research focused on activatable Qdots for drug delivery and imaging [7,8,16]. Typically, when loaded with drugs, these Qdots are put in the “OFF” state with their fluorescence quenched due to energy transfer from the Qdot to the drug or a drug–quencher composite. When the quencher is separated from the Qdot fluorescence is restored [17]. This type of probe reports on drug release events *in situ* by observed changes in fluorescence intensity or wavelength [8,18]. Previous studies have shown that dopamine bound to the surface of a Qdot will quench the Qdot fluorescence due to electron transfer from the electron rich dopamine to the hole in the valence band of the excited Qdot core [19,20,21]. Our previous work has shown that the fluorescence of the dopamine-Qdot conjugate can be restored upon reduction of the disulfide bond by glutathione (GSH) [19]. These activatable Qdots are photo-stable in solution but have limited applications for bioimaging and drug delivery unless functionalized further. These Qdots are then embedded in a polymer network, which helps to minimize Qdot agglomeration and degradation [22,23]. Qdot–polymer composite materials with multiple fluorophores for quantification of relevant molecules at the cellular level are currently being studied [4]. Composite materials are being studied due to their potential applications for topical delivery of drugs and for when single nanosized particles are not desired. These sensors, despite their usefulness, are often difficult and time consuming to synthesize and purify.

To address this shortcoming with existing Qdot–polymer composite sensors, we report an activatable Qdot–chitosan composite gel probe (full probe) to be used for cell tracking and drug delivery. This proof-of-concept study employs Mn^2+^ doped CdS/ZnS core shell Qdots capped with dopamine. The Qdots are fabricated at room temperature using a water-in-oil (W/O) micro-emulsion system, and dopamine is used as a capping agent and quencher of the Qdot fluorescence [20,21,24]. This gives the Qdot activatable properties, since the disulfide bond connecting the dopamine quencher to the Qdot particle is easily broken through reduction by intracellular glutathione [7,8]. The dopamine also acts as a model drug, simulating intercellular drug delivery. These Qdots are then crosslinked to hydrothermally treated chitosan to create a piggyback style, nontoxic, multifunctional probe in a one-step process by using CNBr chemistry. In this cross-linking step, several ligands are also attached to chitosan, including folic acid [5,25,26,27] (FA, targeting motif), polyethylene glycol (PEG dispersing agent) and fluorescein isothiocyanate (FITC, dye for particle tracking). FITC is bound to the chitosan to track the particles in their “OFF” state. Cytotoxicity and interaction of the probe with cells were studied *in vitro* for OVCAR3 (human ovarian cancer), TE71 (murine thymus epithelial), RAW264.7 (murine macrophage) and J774a.1 (murine macrophage). OVCAR3 was selected as it has been shown to overexpress folate receptors, making it an easy target for a folate conjugated particles [28,29]. TE71 cells were chosen as they would represent a typical non-cancerous endothelial cell that a nanoparticle would encounter *in vivo*. J774a.1 monocytes were selected due to both their surface folate receptor expression, and for the preliminary determination of cytotoxic effects of the probe on systemic macrophages encountered *in vivo*. The multimodal functionality and piggyback structure of this probe will allow it to be used in the future by researchers for targeted drug delivery and cell tracking.

## 2. Results and Discussion

### 2.1. Probe Design

**Scheme 1:**
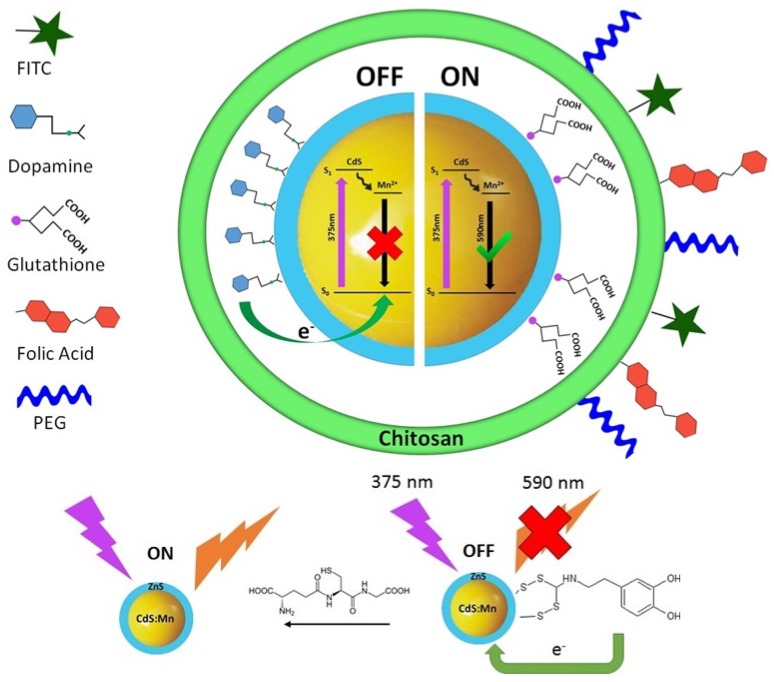
Schematic representation of the overall design of the probe.

The probe was designed to be a nontoxic chitosan nanogel with an embedded activatable Qdot probe to make a composite biosensing material. Core/shell CdS:Mn/ZnS Qdots were used in this study because the ZnS shell renders the Qdots brighter and largely non-cytotoxic [30,31]. As shown in Scheme 1, the basic principle behind the activatability of the quantum dots is the quenching of the Qdot due to electron transfer from dopamine bound to the Qdot [19]. The dopamine is first converted to dopamine dithiocarbamate, which has two sulfur atoms able to form disulfide bonds with the ZnS Qdot shell (Dopamine-Qdot) [19,32,33]. When these quantum dots enter a cell, they will interact with intracellular glutathione where concentrations can reach 10 mM [34]. Glutathione is able to perform ligand exchange with the dopamine on the Qdot surface [19]. The release of dopamine from the Qdot surface restores the Qdot fluorescence. This activatability was incorporated to allow for activated drug release and reporting on this event upon internalization by the cell. Previous studies have shown that some cancer cells have elevated levels of glutathione, theorized to be a response to increased free radical concentrations [34,35,36]. This makes glutathione mediated activation a viable route for triggering drug release events.

Hydrothermally treated chitosan particles were chosen for functionalizing the Qdots because they are biocompatible, soluble in water, and contain a primary amine group that can be exploited for ligand attachment [37,38,39]. FITC was attached to the chitosan to be used for particle tracking purposes as its fluorescence is always “ON”, unlike the Qdot. The chitosan was cross-linked around the Qdots and to the targeting ligand (FA) to form the full probe. The probe was synthesized with folic acid (+) FA and without folic acid (−) FA CNBr chemistry, which binds primary amine groups to alcohol groups, and was used as a cross-linker. CNBr was chosen to cross-link the components because cross-linking can be completed in one step. In addition, CNBr reacts with the widely available amine and alcohol groups in chitosan, folic acid, PEG, and dopamine. After cross-linking, the gel probe was washed with ethanol to remove any unbound components.

### 2.2. Infrared Spectroscopy

**Figure 1:**
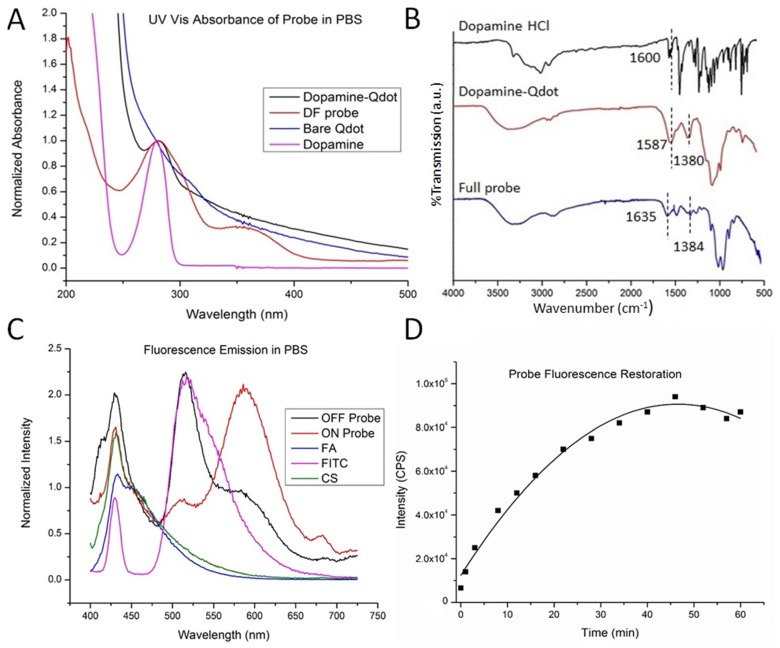
(**A**) UV-vis absorbance of dopamine HCl, bare CdS:Mn/ZnS Qdots, dopamine-Qdot conjugate, and the full probe. The peak at 278 nm is attributed to absorption by dopamine. This peak is evidence of dopamine remaining bound to the Qdot surface after washing and coating with chitosan; (**B**) FTIR spectra of dopamine HCl, dopamine-Qdot conjugate, and the full probe; (**C**) Normalized fluorescence emission spectra of the full probe in the “OFF” state, the full probe in the “ON” state (Probe + 10 mM glutathione (GSH)), folic acid (FA), Fluorescein isothiocyanate (FITC), and hydrothermally treated chitosan (CS) in PBS buffer obtained with 375 nm excitation; (**D**) Fluorescence intensity of Qdots in PBS over time after addition of 10 mM GSH. Full restoration of Qdots is observed 40 min after addition of GSH. This shows the extent to which fluorescence can be restored for the probe. A polynomial function was fitted to the data to determine the linearity of fluorescence restoration.

Due to the surfactant based Qdot synthesis, multiple washing steps were performed on the Qdots. Infrared spectroscopy was employed to determine the presence of bound dopamine and chitosan to the Qdots after washing and lyophilizing (Figure 1A). The peaks found at 1587 and 1380 cm^−1^ in the dopamine-Qdot conjugate, and the 1635 and 1384 cm^−1^ peaks observed for the full probe can be attributed to the stretching of C–C bonds in the aromatic ring. These bands are not observed in the FTIR spectra of bare Qdots (not shown), indicating that dopamine is present on the Qdot surface after washing. C–O stretching vibrations of chitosan were observed for the full probe at 1071 cm^−1^, as reported in literature [40,41]. This absorption masks the absorption of dopamine hydroxyl groups, since chitosan is present at higher concentrations in the probes. Absorption peaks characteristic of the isocyanate bond between FITC and chitosan were observed at 1562, 1395, and 1002 cm^−1^ (Figure S1) [42]. These observations confirm the presence of FITC after washing.

### 2.3. UV-Vis Spectroscopy

The optical properties of the probe were characterized by UV-Vis absorption and fluorescence spectroscopy. Figure 1B shows the UV-Vis absorbance spectra of the probe and Qdots acquired with water as the solvent. An absorption peak was observed at 278 nm that was assigned to dopamine [43]. This peak was found in both spectra of the dopamine–Qdot conjugate and the full probe. These spectra show that dopamine remains on the Qdot surface after rigorous washing. The strength of the interaction is due to the potential for dopamine dithiocarbamate to form two disulfide bonds with the Qdot surface in a bidentate fashion [32]. This makes the Qdot–dopamine bond more stable and helps prevent premature displacement from the Qdot surface by media proteins.

### 2.4. Fluorescence Spectroscopy

Fluorescence emission spectra of the full probe and the individual constituent components were collected under 375 nm excitation with buffer as the solvent (Figure 1C). The emission peak at 450 nm is attributed to a combination of chitosan and folic acid fluorescence [44]. The peak at 490 nm is due to FITC bound to chitosan, and the peak observed at 590 nm is the Qdot fluorescence. All of these emission peaks are present in the spectra of the full probe indicating that all the components have been successfully bonded as any unbound reagent would be washed off. The full probe was treated with 10 mM GSH to turn it to the “ON” state; this is to simulate the environment of a cancer endosome. Ten millimolar GSH was then added to the probe, and fluorescence spectra were collected at 5 min intervals [45]. The concentration of GSH in blood plasma of humans was found to be less than 1 mM in previous studies [46]. The change in intensity of the emission peak at 590 nm was then plotted as a function of time (Figure 1D). This restoration of fluorescence plateaued 40 min after GSH addition.

### 2.5. Electron Microscopy

**Figure 2:**
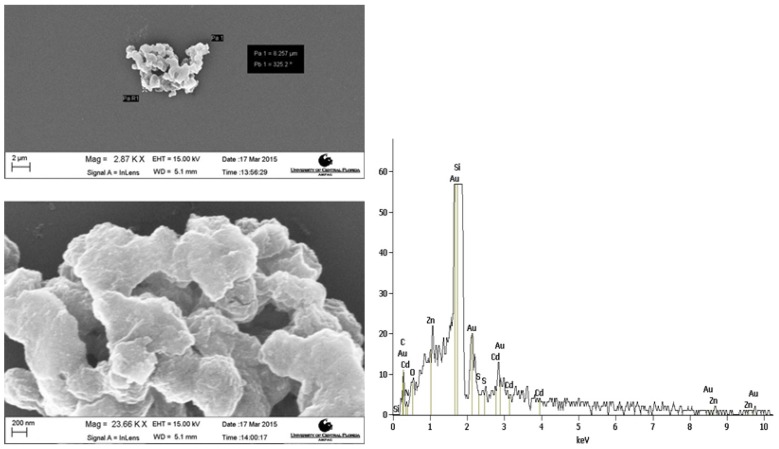
Scanning Electron Microscopy (SEM) images and Energy Dispersive Spectroscopy (EDS) spectrum of the gel probe. The full probe is composed of small particles that are embedded in a cross-linked chitosan matrix. The particle pictured above could possibly be multiple aggregated particles. The EDS spectrum shows small peaks for cadmium and zinc, which confirm the presence of Qdots in the chitosan matrix.

**Figure 3:**
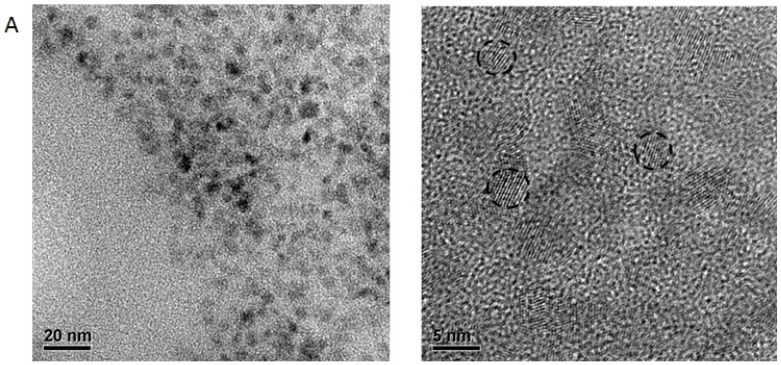

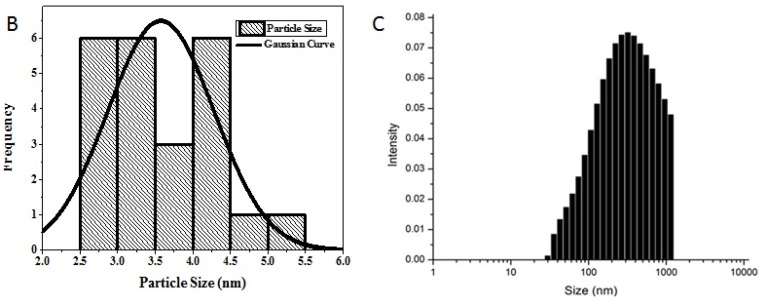
(**A**) Transmission Electron Microscopy (TEM) images of the bare CdS:Mn/ZnS Qdots. Three Qdots are circled for clarity in the image on the right; (**B**) The Qdot size distribution as determined by the observation of the lattice planes of 23 of the most clearly observed individual Qdots. Data were taken from multiple images (not shown). A normal distribution was fitted to the data to determine the average Qdot size; (**C**) Size distribution of the full probe dispersed in water as measured by DLS. The size scale is logarithmic. The apparent cutoff on the right hand side of the histogram is due to the absence of particles with diameter greater than 2000 nm.

Scanning Electron Microscopy (SEM) analysis was used in conjunction with SEM Energy Dispersive Spectroscopy (EDS) to analyze the size and morphology of the full probe and show the presence of Qdots in the chitosan/Qdot composite. Figure 2 shows a SEM image of the probe. The Qdots were too small to be resolved, however, EDS shows the presence of cadmium. The hydrothermal treatment and filtration of the chitosan prior to mixing with the quantum dot shows mostly small chitosan particles remaining. A wide range of sizes of the full probe was observed with the average size falling near 1 mn in diameter. Many large particles were observed due to aggregation of the particles while coating the SEM grid, and the high efficiency of the CNBr cross-linking of chemistry. Transmission Electron Microscopy (TEM) analysis performed on bare Qdots shows monodisperse spheres with diameters ranging from 3.5 to 5.0 nm (Figure 3A). The TEM images were analyzed to find the particle distribution (Figure 3B). Single Qdots were identified by their characteristic lattice lines observed in the TEM. Dynamic Light Scattering (DLS) data on the full probe (Figure 3C) showed an average size of 380 nm following sonication, which is probably more representative of the probe in a moderately dispersed state compared to the SEM results.

### 2.6. Cytotoxicity

**Figure 4:**
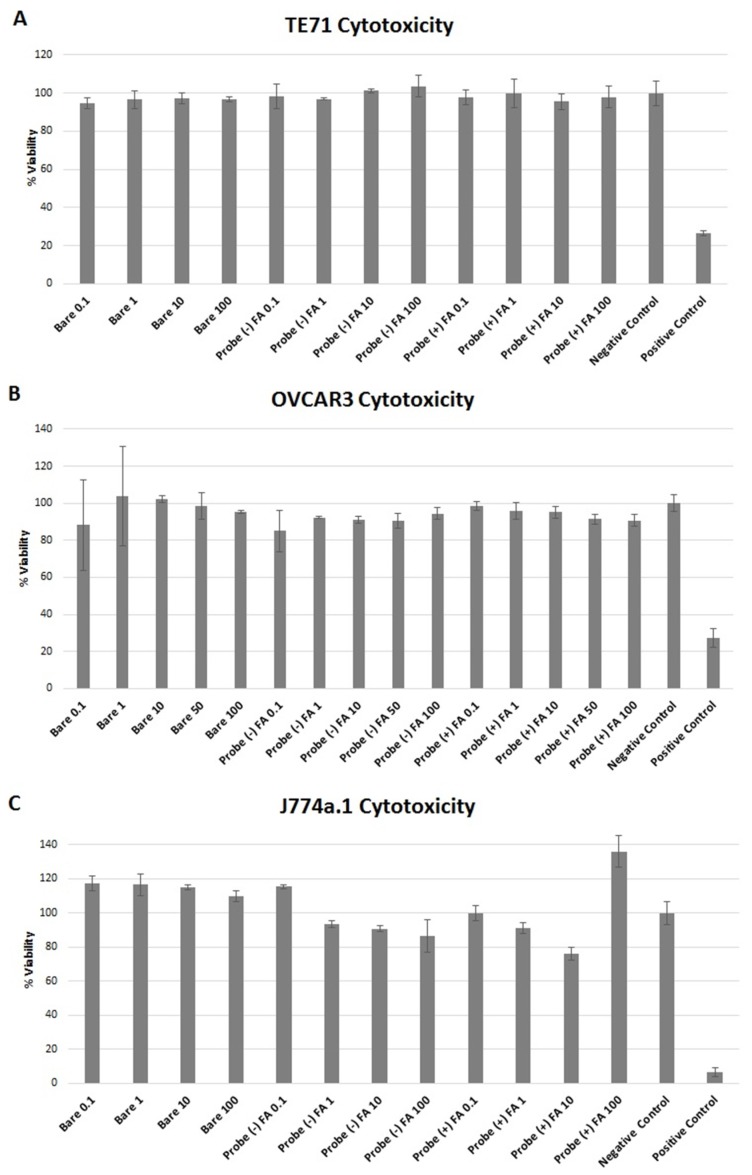
(**A**) Observed cytotoxicity towards TE71; (**B**) OVCAR3; and (**C**) J774a.1 macrophage cells after 24 h incubation with bare Qdots and the full probe as measured by MTS assay. Bare Qdots and full probe with folic acid (+) FA and without folic acid (−) FA bound to the chitosan at 100, 10, 1, and 0.1 μg/mL Qdot concentration. The growth control (negative) is untreated cells in media. The positive control for cell death was completed by adding water to the cells. The cytotoxicity data are plotted relative to the negative control, which was assigned to have 100% viability. Error bars are standard deviation of the mean.

To test the biocompatibility of the full probe and its components, bare Qdots as well as the full probe were applied to OVCAR3, TE71, J774a.1, and RAW264.7 cells in a 96 well plate, and a cell proliferation assay (MTS assay) was performed after 24 h incubation. Macrophages are known to rapidly internalize large particles of dust or bacteria cells. For a Qdot based probe to be applicable for biological research, it would need to either bypass internalization by macrophages and/or be nontoxic to them as they are present in most multicellular organisms. MTS assay (formazan dye) was used to assess the toxicity of the full probe with these three cell lines. MTS assay showed both bare Qdots and the full probe to be nontoxic to OVCAR3, TE71, and J774a.1 macrophage cells even at the highest concentration tested, 100 mg/L (Figure 4). To cross validate that the Qdots were not interacting with the MTS dye, an Alamar blue cell proliferation assay (Resazurin dye) was completed for the RAW264.7 macrophages at the same concentrations of Qdot and full probe (Figure S2).

The Alamar blue assay showed minimal cytotoxicity to the RAW264.7 macrophages, consistent with the MTS assay. The mild toxicity for J774a.1 and RAW2647 macrophages was thought to be due to increased internalization of the particles by these cell lines, and this is assessed later. The toxicity data obtained from the MTS assay are consistent with previous studies that have correlated various doses of CdTe/CdSe Zn/S core–shell quantum dots with a variety of toxic response markers specific to cadmium-based nanomaterials [30,47]. The observed increase in viability for the highest doses of +FA probe for the J774a.1 cells could be due to increases in mitochondrial reductase activity as recently reported [48,49,50].

### 2.7. Cell Uptake

**Figure 5:**
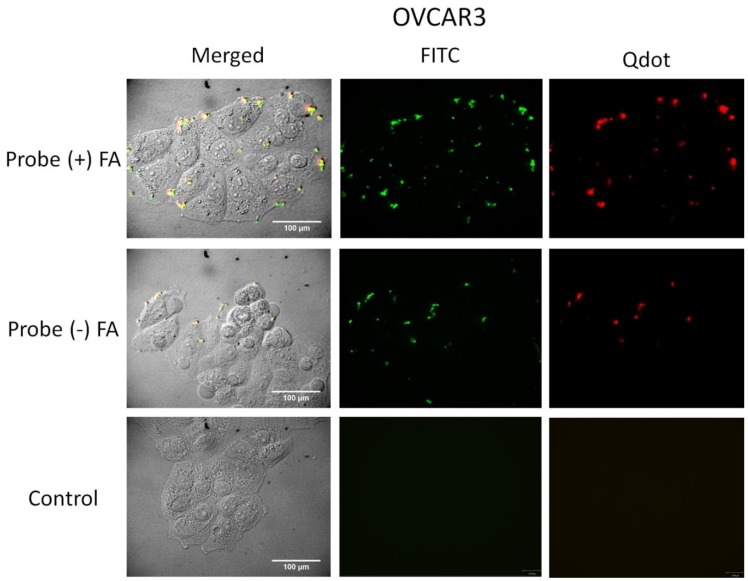
Confocal microscopy images of OVCAR3 cells incubated with the full probe with (+) FA and without folic acid (−) FA, and with media without probe (control). The right column images display the fluorescence of the Qdots only. The middle column images show just the fluorescence from FITC. The left images are phase images merged with Qdot and FITC fluorescence images. Qdots fluorescence is seen co-localized with fluorescence of chitosan bound FITC. Increased binding of the probe to OVCAR3 cells was observed with samples containing attached folic acid (Probe (+) FA). The full Z-stack images can be viewed in the supplemental section (Figures S3–S6).

**Figure 6:**
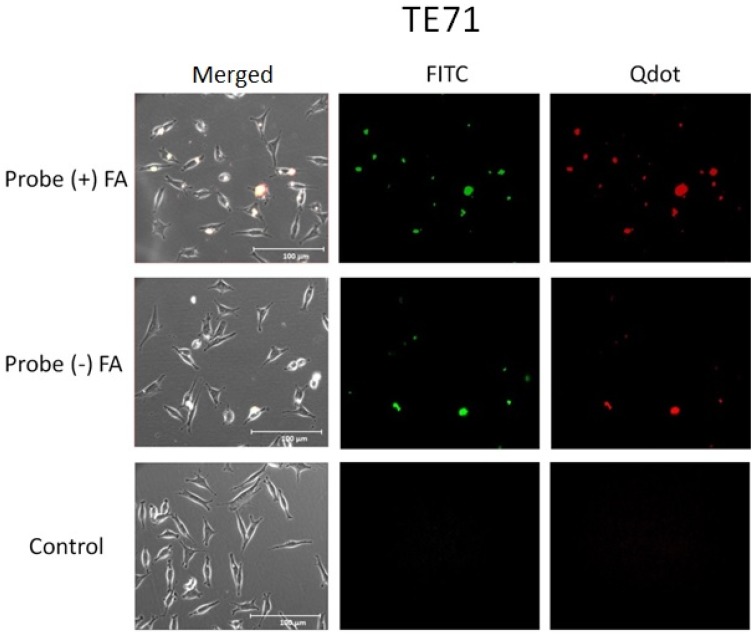
Fluorescence microscopy images of TE71 cells incubated with the probe with bound folic acid (+) FA and without folic acid (−) FA, and with media without probe (control). The left images are phase images merged with the fluorescence images. The middle images are only the FITC fluorescence. The right images are only the Qdot fluorescence. Only a small increase in binding of the full probe to the TE71 cells was seen from the probe (+) FA. This is most likely because TE71 does have some folate receptors but has not been shown to overexpress them.

**Figure 7:**
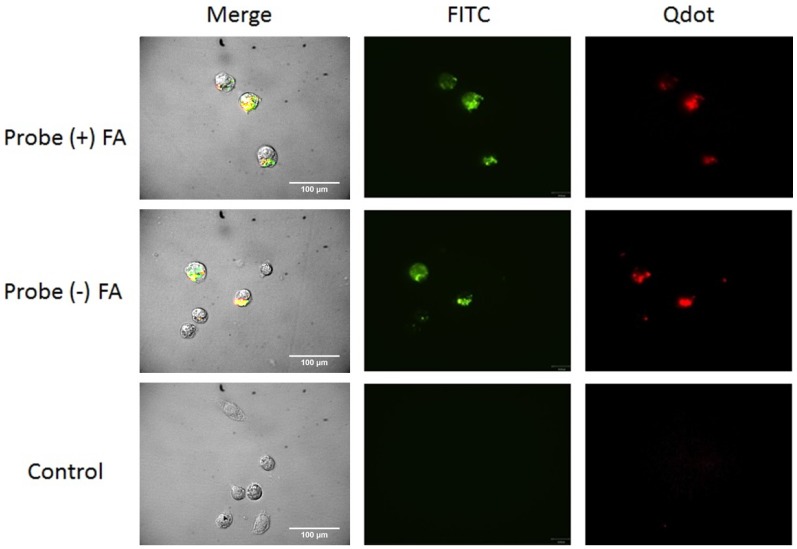
Confocal microscopy images of J774a.1 macrophage cells incubated with the full probe with (+) FA and without folic acid (−) FA, and with media without probe (control). The left images are merged phase and fluorescence images. The middle image are only FITC fluorescence and right images are only Qdot fluorescence. A large amount of the full probe was bound and some internalized by the cells. Macrophages are known to nonspecifically internalize particles which contribute largely to internalization of the probe by these cells.

Preliminary cell uptake studies were performed to study the interaction of the particles with cells. (Figure 5, Figure 6 and Figure 7). Studies have shown that some cancer cells such as OVCAR3 overexpress folate receptor proteins on their cell membranes [28,29]. Folate is essential for DNA synthesis and is used rapidly by fast dividing cancer cells. Overexpression of these receptors has been used as a biomarker for detecting cancer cells [26,51,52]. TE71 were used as a normal control cell line that does not overexpress folate receptors. J774a.1 murine monocytes were used as a cell line representative of systemic non-differentiated macrophages. These express folate receptors as well. Confocal images of the cells show that the full probe remained mostly bound to the cell surface. This observation is consistent for all cell lines, while some was internalized by the macrophages (Figure 7). The control cell lines not over-expressing folate receptors internalized similar amounts of folate (+) and folate (−) probe as expected (Figure 6 and Figure 7). Both OVCAR3 and J774a.1 cells were observed to bind more of the probe that included conjugated folate (+ FA) on the cell surface. This effect was more pronounced in OVCAR3 than the other cell lines due to the increased number of receptors on the surface of OVCAR3 cells. The amount of probe bound in each circumstance is determined by the total Qdot and FITC fluorescence assessed visually.

We noted significant internalization of the full probe by J774a.1 monocytes through confocal microscopy imaging for both the folate positive and folate negative probe (Figure 7). The J774a.1 mouse monocyte cells are known to express Fc gamma receptor sites on the extracellular domain of the cell membrane [53]. This folate receptor expression is a prime contributor to the observed macrophage internalization of folate positive probe (+FA) via folate receptor specific pathway. Additionally, nonspecific endocytotic/phagocytotic pathways may be a contributor to the internalization of the folate negative probe (−FA). This pathway specific sensitivity is attributed to a variety of properties, such as size, shape, charge, and surface functionality of the probe [54,55,56].

**Figure 8:**
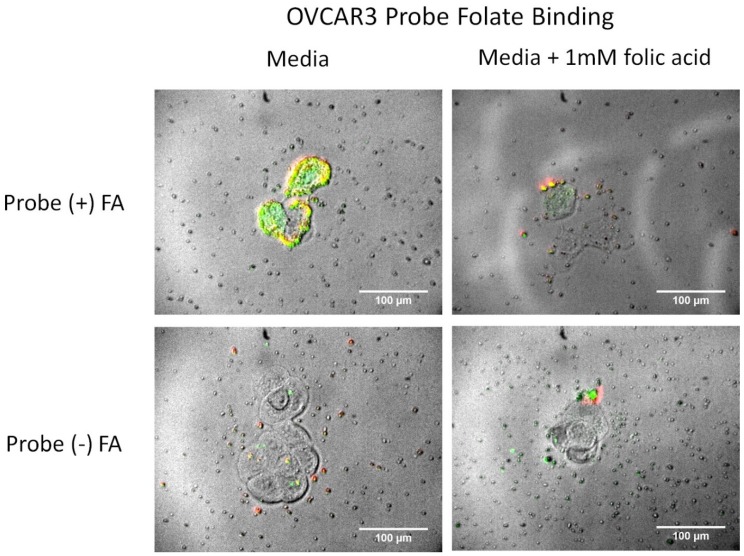
Fluorescence images of OVCAR3 cells after incubation with the full probe. (**Left**): Cells were incubated with regular DMEM media. (**Right**): Cells were incubated with media that had extra folic acid added (1 mM). The high concentration of free folic acid binds to the folate receptors blocking them from interacting with the folic acid bound to the probe.

The dependency of uptake on binding to folic acid receptors is shown in Figure 8 for OVCAR3 cells. The dependency of folate receptor interaction on the observed total full probe binding was analyzed through fluorescence and confocal microscopy. Cells were incubated with the full probe either with or without extra folic acid (1 mM) added to the media. The presence of a large concentration of free folic acid in media has been previously shown to decrease the uptake of folate-conjugated particles since access to folate receptors becomes compromised as they become saturated with free folic acid [57]. The fluorescence intensity of the probe bound to cells incubated in folate media was much less than cells incubated with folate free media (Figure 8). These results suggest that folate receptors play a large part in the binding of the probe to the OVCAR3 cells.

## 3. Experimental Section

### 3.1. Materials

Cadmium acetate dehydrate, manganese (II) acetate tetrahydrate, glutathione (GSH), and Dioctyl sodium sulfate (AOT) were all purchased from Acros Organics (Morris Plains, NJ, USA). Sodium Sulfide, zinc acetate dihydrate, dopamine hydrochloride, cyanogen bromide (CNBr), and low molecular weight chitosan were purchased from Sigma-Aldrich (St Louis, MO, USA). Fluorescein isothiocyanate (FITC), heptane, dimethylsulfoxide (DMSO), trimethylamine, methanol, ethanol, hydrochloric acid, and folic acid were purchased from Thermo Fisher Scientific (Waltham, MA, USA). Acetonitrile was obtained from Alfa Aesar (Ward Hill, MA, USA). Nanopure water was obtained from a Barnstead Nanopure Diamond Model # D11911 (Thermo Scientific, Waltham, MA, USA). All materials were used as received at room temperature and without further purification. All solvents used were reagent grade and used as received.

### 3.2. Synthesis of Dopamine Coated CdS:Mn/ZnS Quantum Dots

The Qdots were prepared from a micro-emulsion method using previously established protocol [8,58] with additional modifications. Stock solutions of 266 mg cadmium acetate dihydrate and 4.9 mg manganese acetate tetrahydrate in 10 mL water, 257.5 mg sodium sulfide in 5 mL water and 285.25 g zinc acetate dihydrate in 5 mL water were prepared. One hundred and seventy-five milliliters of a heptane and 15.49 g AOT solution was prepared and separated into three flasks, one with 25 mL and two with 75 mL each. Then, 0.9 mL of the cadmium solution was added to the 25 mL flask, and 2.7 mL of the sulfur and zinc were added to the 75 mL flasks. The flasks were covered and magnetically stirred for 1 h. The cadmium flask was then slowly added to the sulfur flask and allowed to stir for 15 min. The zinc flask was added dropwise (2–3 mL/min) to the cadmium/sulfur flask to provide the ZnS shell layer. The Qdots were then covered and left to stir a week before use. Dopamine dithiocarbamate was synthesized by reaction of 104 mg of dopamine hydrochloride with 160 μL of CS_2_, 200 μL methanol and 50 μL of Et_3_N [32,33]. Three milliliters of the Qdots in micro-emulsion was then reacted with dopamine dithiocarbamate for 3 min. The dopamine–Qdots were washed five times in ethanol to remove any unbound dopamine.

### 3.3. Synthesis of Chitosan Coated Qdots

Chitosan was first depolymerized by hydrothermal treatment as previously described [59]. Three hundred milligrams of chitosan was dissolved in 30 mL of 1% hydrochloric acid and transferred to a Teflon container, which was placed sealed in a steel container. Samples were heated at 150 °C for 90 min. The chitosan was then filtered with a 0.22 μm syringe filter and dialyzed for three days while changing the dialysis water every 8 h to bring the pH to neutral and remove the chlorine ions. The chitosan was then filtered again with a 0.22 μm syringe filter. The sample was then frozen and lyophilized for use as coating. Chitosan coating for the probe was accomplished by mixing solutions of 3 mg of lyophilized hydrothermally treated chitosan, 0.6 mg folic acid, 0.6 mg FITC, and 1.2 mg PEG in 3 mL PBS for 1 h. The same coating solution was prepared without folic acid for the (− FA) probe. This coating was then mixed with the dopamine–Qdot conjugate in the sonicator for 1 h. Three milligrams of CNBr in 15 μL acetonitrile was added and allowed to react for 20 s for cross-linking the polymer and Qdots. The coated Qdots were then washed 4 times with ethanol to remove any unbound coating and dye.

### 3.4. FTIR Experiments

The FTIR spectra of the full probe and components were collected using a PerkinElmer Spectrum 100 Series ATR FT-IR Spectrometer (PerkinElmer, Waltham, MA, USA). All samples were frozen in DI water or DMSO and lyophilized (FreeZone 4.5 L Freeze Dry System, Labconco, Kansas City, MO, USA) to dry powder prior to analysis.

### 3.5. Solution Spectroscopy

UV–Visible absorbance spectroscopy was employed using a Cary 300 UV-Vis Spectrophotometer (Agilent, Santa Clara, CA, USA). The solvent used was 1× PBS in a 1 cm quartz cuvette. Concentrations of the samples were standardized to adjust for batch-to-batch differences in concentration by adjusting their absorbance at 375 nm to 0.05. Fluorescence spectra were collected on a NanoLog Fluorescence Spectrophotometer (SPEX, Jobin Yvon Horiba, Edison, NJ, USA). The probe was treated with 10 mM GSH to simulate the effect of GSH binding inside the cell to turn the probe ON. The excitation wavelength used was 375 nm as it has maximum absorption by the Qdots.

### 3.6. Scanning Electron Microscopy (SEM)

Samples for SEM were dispersed in water and spin coated onto a silica wafer. They were then gold coated for one minute. Samples were analyzed by a Zeiss ULTRA-55 FEG SEM (Zeiss, Oberkochen, Germany) equipped wit/h a Noran System 7 EDS with Silicon Drift Detector (Thermo Scientific, Madison, WI, USA).

### 3.7. Dynamic Light Scattering (DLS)

DLS measurements were preformed using a PDDLS/Cool/Batch 40 T Precision Detector (Chia Yun Instrument Inc., Taipei City, Taiwan). Data was processed using Precision Deconvolve software (Precision Detectors Inc., Bellingham, MA, USA).

### 3.8. Cytotoxicity

TE71 (mouse thymus epithelial ATCC), OVCAR3 (human ovarian cancer ATCC), and J774a.1 (BALB/c mouse monocyte cell line, ATCC) cells were grown in Dulbecco’s modified Eagles’s medium (DMEM, Corning, NY, USA) with 10% heat-inactivated fetal bovine serum (20% for OVCAR3 cells). The cells seeded at a density of 40,000 cells per well onto a 96-well plate (Cellstar, Sigma-Aldrich, St. Louis, MO, USA). Cells were then incubated 24 h and washed with PBS. The cells were then treated with Qdots and probe for 24 h, after which a background absorption spectra of the plate was taken at 490 nm (BioTek ELx808 absorbance plate reader (BioTek Instruments, Winooski, VT, USA) and the MTS dye (CellTiter 96 Aqueous One Solution Cell Proliferation Assay, Fisher Scientific (Thermo Fisher Scientific, Waltham, MA, USA) was applied. Cells were incubated 1–2 h at 37 °C and 5% CO_2_, and the absorption of the MTS dye was again taken at 490 nm. The background absorption of the particles was subtracted from absorption of the dye. Percentage of viability was calculated by dividing the samples by the absorbance of MTS dye for cells in growth media.

For Alamar blue assay, macrophages were seeded in a 96 well plate at the density of 2 × 10^4^ cells/well using DMEM medium (10% FBS) and incubated at 37 °C and 5% CO_2_ for overnight growth. Then, the medium was replaced by fresh medium before adding Qdots and full probe samples. After 24 h of incubation at 37 °C and 5% CO_2_, the medium was again replaced and the Alamar blue reagent was added. The plate was incubated for an additional 3 h and the fluorescence was read at 590 nm emission (550 nm of excitation). Cell viability was calculated by dividing the fluorescence for samples by the fluorescence for cells in growth media (negative control).

### 3.9. Cell Uptake Studies

TE71, OVCAR3, and J774a.1 macrophage cells were cultured in DMEM/F-12 media (Corning) with 10% heat inactivated FBS (OVCAR3 20%) in 25 cm^2^ treated cell culture flasks (Cellstar) at 37 °C and 5% CO_2_. OVCAR3 cells were cultured in the same way while using 20% heat inactivated FBS. Cell viability was determined using Trypan blue dye exclusion assay (Thermo-Fisher Scientific, Waltham, MA, USA) and cells were counted with a hemocytometer (Hausser Scientific Partnership, Horsham, PA, USA) after trypsinization. Only cell cultures with viability greater or equal to 95% were used. Cells were seeded onto a petri dish (Corning 35 mm × 10 mm tissue-culture treated) at a density of 100,000 cell/cm^2^. The dish was incubated for 24 h at 37 °C and 5% CO_2_. The cells were washed 5 times with HBSS and the media was replaced with the sample in DMEM/F-12 with 10% FBS at a concentration of 10 mg/L (Qdot concentration). Cells with no probe added were imaged as a control. The cells were incubated for 3 h and then washed five times with PBS to remove any particles that were not bound or internalized by the cells. The cells were then fixed with 4% paraformaldehyde (Electron Microscopy Sciences, Hatfield, PA, USA) before imaging and washed two more times. Approximately 15–20 fields of view were assessed with 10 cells per field, accounting for nearly 75% of the total occupied surface area. Average representative images were taken (Figure 5, Figure 6, Figure 7 and Figure 8) to visually assess overall binding/uptake of probe. This experiment was repeated three times with independently synthesized samples, all yielding similar results.

### 3.10. Fluorescence Spectroscopy

Fluorescence images were taken using Olympus IX71 epiluminescence microscope with a LUCPlanFLN 60×/0.70 ph2 objective lens (/0.1–1.3/FN22) (Olympus, Tokyo, Japan). Images were captured by Andor Zyla sCMOS (DG-152V-C1E-FI) camera (Andor, Oxford Instruments, Belfast, UK) with Micro-Manager 1.4 version software. Mercury lamp was used as an excitation source for FITC and Qdot fluorescence. For FITC images, excitation, dichroic mirror and emission filters (491/10 nm, 510 dclp, 514 ref, respectively) were used. For Qdot images, excitation dichroic mirror and emission filters were used respectively 360/40, 405lp, 585/20. The images were overlaid with ImageJ software (NIH, Bethesda, MD, USA).

### 3.11. Confocal Imaging

Confocal images were acquired using a Zeiss Axioskop 2 plus upright microscope (Zeiss, Oberkochen, Germany) with a 40× water immersion objective. Fluorescence pictures were captured with a Hamamatsu ORCA-Flash4.0 camera (Hamamatsu Photonics, Tokyo, Japan) using PerkinElmer Volocity software (PerkinElmer, Waltham, MA, USA). Qdot pictures were taken with 365/10 nm excitation filter using a fluorescence lamp (X-Cite 120Q) with 607/45 nm emission filter (Excelitas Technologies, Waltham, MA, USA). Excitation of FITC was accomplished using 488 nm Melles Griot laser (643-PERYB-AO2) with a 525/50 emission filter (CVI Laser Optics, Albuquerque, NM, USA). Samples were scanned a total 50 μM thickness with a 2 μM step size. ImageJ software (NIH, Bethesda, MD, USA) was used to further process the images.

## 4. Conclusions

A multifunctional chitosan–quantum dot (Qdot) composite based nanogel probe was designed and synthesized. A one-step cross-linking of multiple components with CNBr chemistry was described. The design of the probe is novel in that it is based on a piggyback system employing activatable Qdots and chitosan gel for multimodal applications. Systematic spectroscopic and microscopic studies were completed to characterize the activatable (“OFF”/“ON”) biosensing properties, drug delivery (using dopamine as a model drug as well as Qdot fluorescence quencher), and folic acid mediated cancer targetability. The probe was found to be nontoxic and could readily bind to cancer cells, normal cells, and macrophages. Folic acid linked to the chitosan was observed to enhance binding in OVCAR3 cells due to elevated folate receptor expression. Additional experiments on drug delivery and cellular uptake are being carried out with an anti-cancer drug and will be published in the near future.

## Supplementary Materials

Supplementary materials can be accessed at: http://www.mdpi.com/2079-4991/5/4/2359/s1.
