# Supplementary material for: Non-Cytotoxic Quantum Dot–Chitosan Nanogel Biosensing Probe for Potential Cancer Targeting Agent
Source: Nanomaterials (Basel). 2015 Dec 18;5(4):2359–79. doi: 10.3390/nano5042359 (PMC5304800; doi:10.3390/nano5042359)
Supplement: Supplementary file 1 [file nanomaterials-05-02359-s001.pdf]

Supplementary Materials

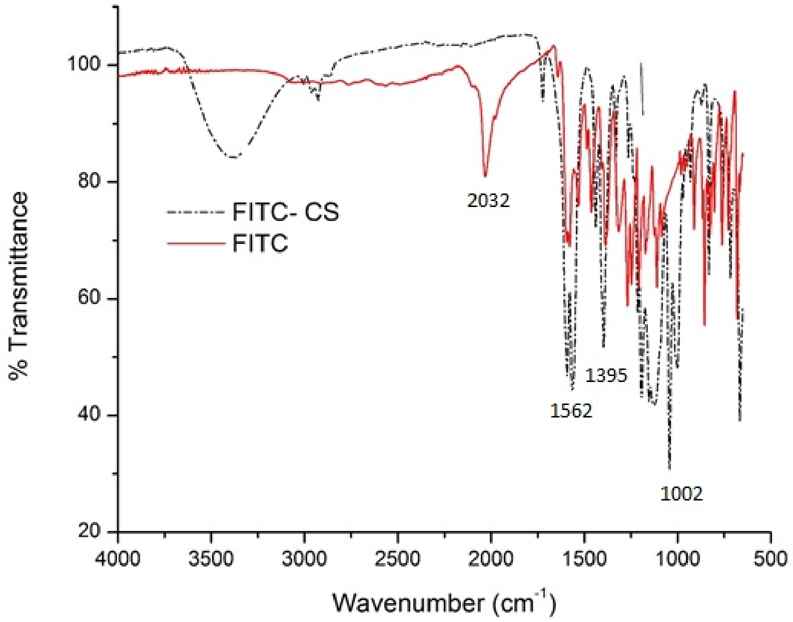

Figure S1. FTIR spectra of Fluorescein Isothiocyanate (FITC) and lyophilized FITC-chitosan gel. The chitosan gel particles were reacted with FITC washed with ethanol. The peak at 2032 cm^{-1} is attributed to C=N stretching in the isothiocyanate group of FITC. Once this group has formed an isourea bond, the stretching is lost. The peaks at 1562, 1395, and 1002 cm^{-1} are characteristic of a thiourea bond [1].

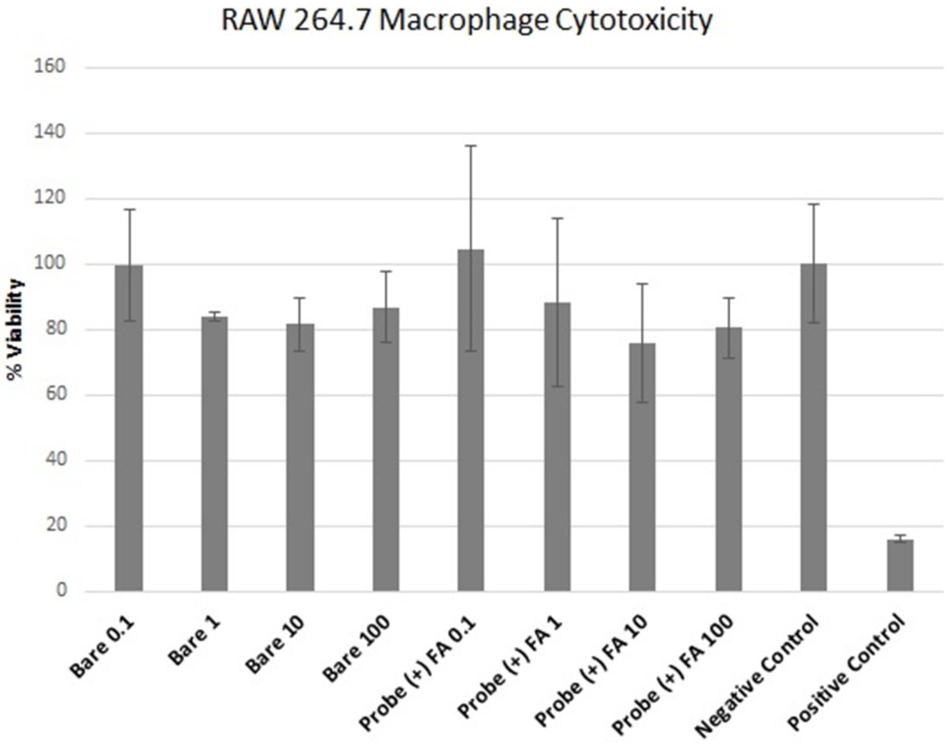

Figure S2. Percentage viability of RAW 264.7 macrophage after treatment with bare Quantum dots (Qdots) and the probe. Viability was calculated by Alamar blue assay, the increase in fluorescence at 590 nm is correlated with cellular activity.

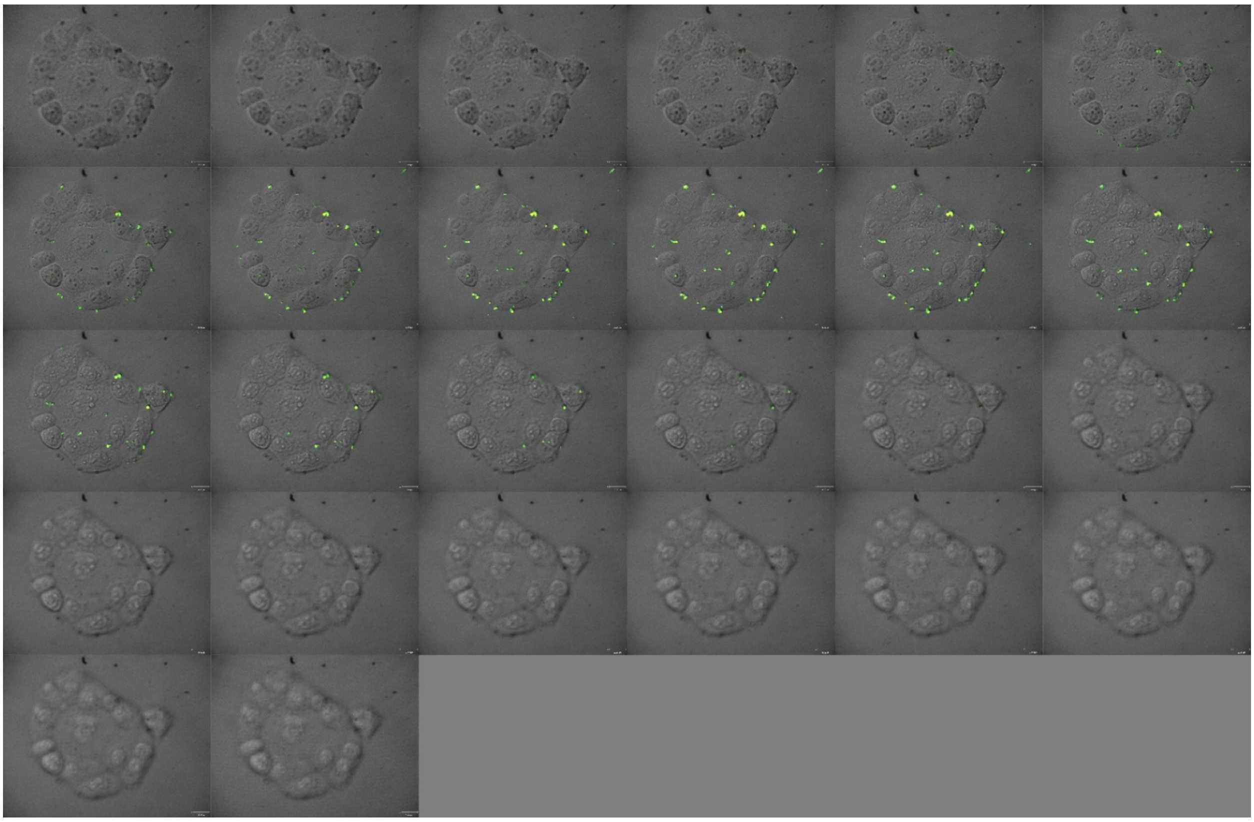

Figure S3. Merged Z-stack confocal image of OVCAR3 cells incubated (+) FA probe. Top left image is the top progressing to the bottom right at 2 μm per image.

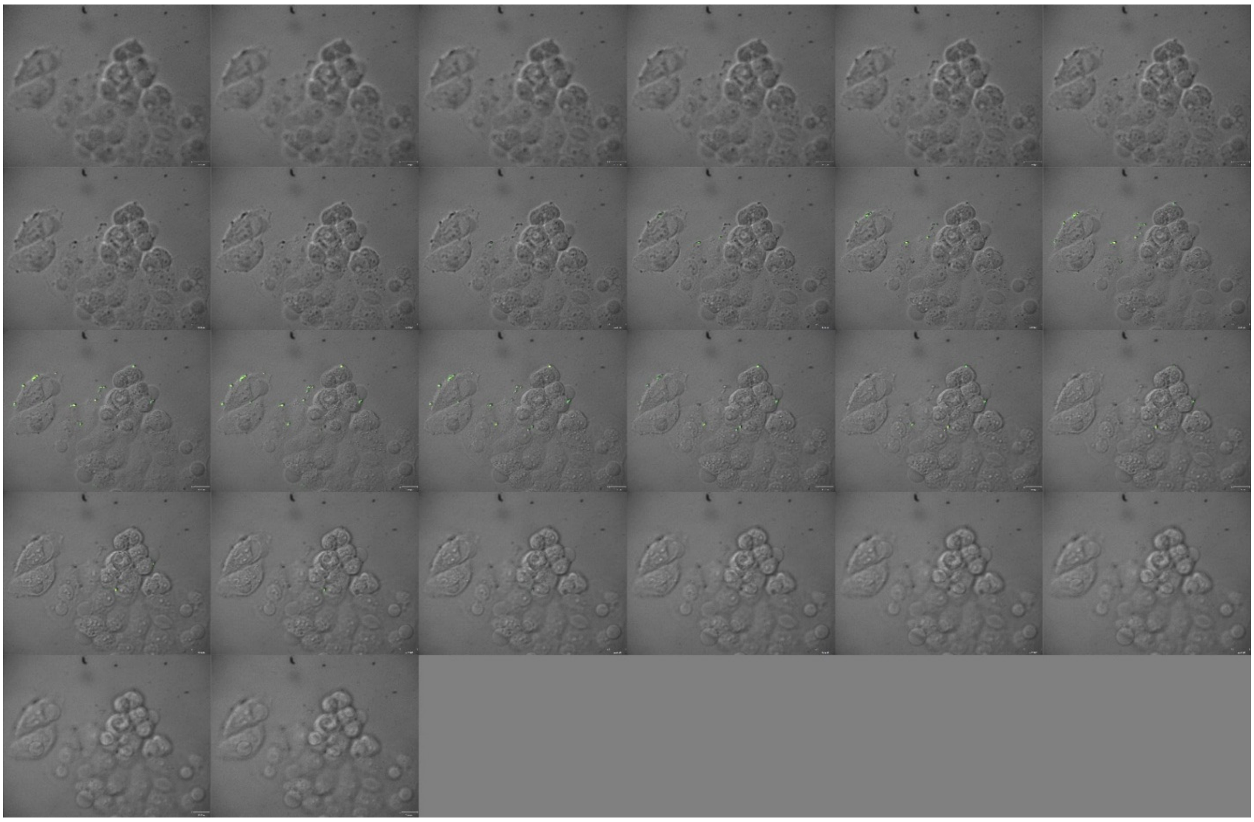

Figure S4. Merged Z-stack confocal image of OVCAR3 cells incubated (−) FA probe. Top left image is the top progressing to the bottom right at 2 μm per image.

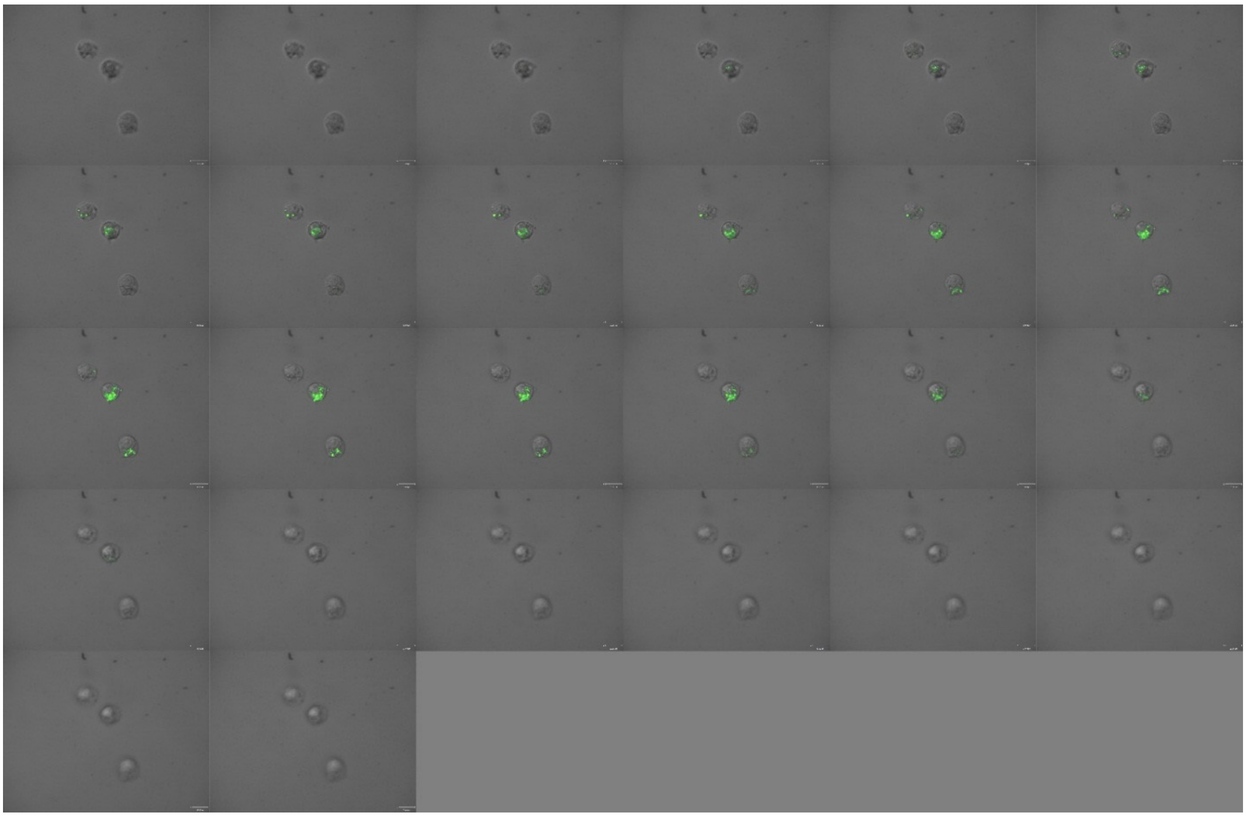

Figure S5. Merged Z-stack confocal image of J774a.1 cells incubated (+) FA probe. Top left image is the top progressing to the bottom right at 2 µm per image.

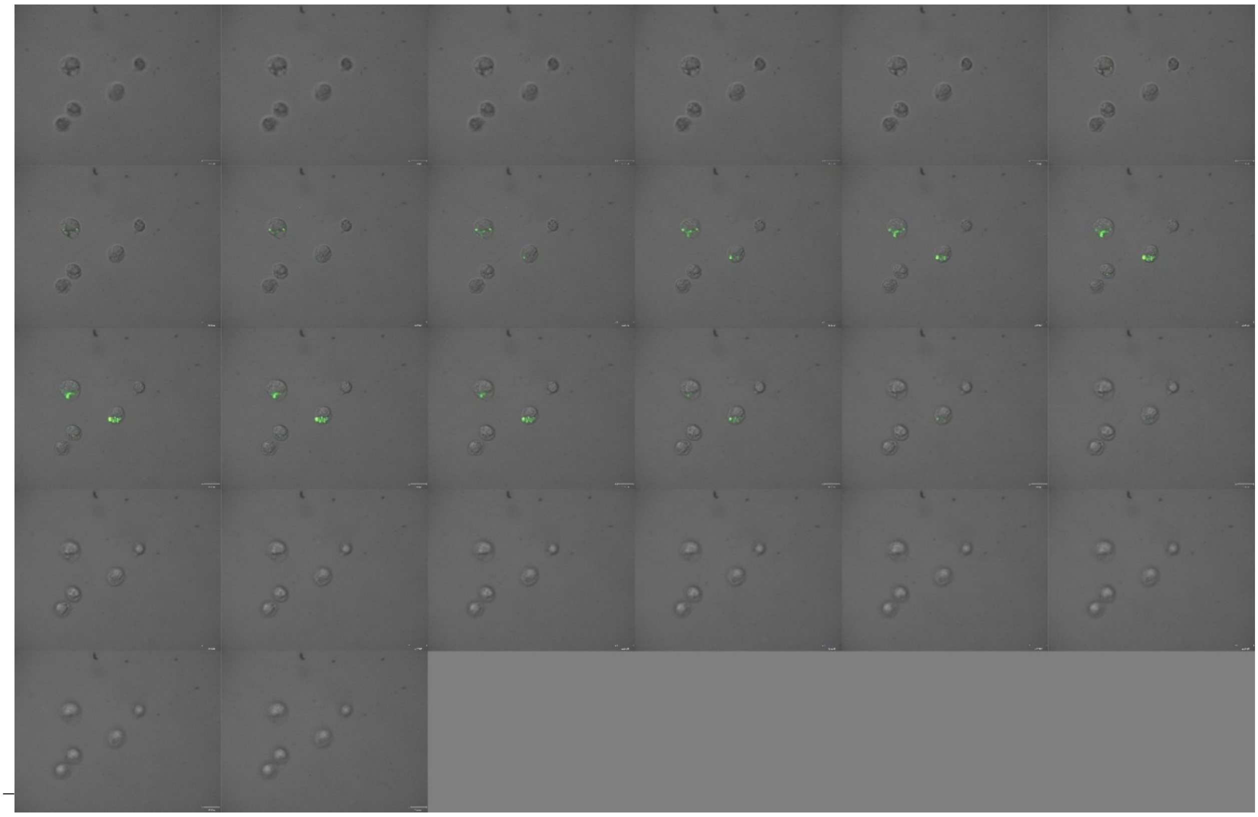

Figure S6. Merged Z-stack confocal image of J774a.1 cells incubated (−) FA probe. Top left image is the top progressing to the bottom right at 2 µm per image.

References

1. Studer, K.; Decker, C.; Beck, E.; Schwalm, R.; Gruber, N. Redox and photoinitiated crosslinking polymerization. *Prog. Org. Coat.* **2005**, *53*, 126–133.

© 2015 by the authors; licensee MDPI, Basel, Switzerland. This article is an open access article distributed under the terms and conditions of the Creative Commons Attribution license (http://creativecommons.org/licenses/by/4.0/).
